# Reflections from the Janus face of gibberellin in legume nodulation

**DOI:** 10.1093/jxb/ery102

**Published:** 2018-04-09

**Authors:** Peter J Davies

**Affiliations:** Section of Plant Biology, School of Integrative Plant Sciences, Cornell University, Ithaca, NY, USA

**Keywords:** DELLA, GA_3_, gibberellin, nodulation, pea, *Pisum*, *Rhizobium*

## Abstract

This article comments on:

**McAdam EL, Reid JB, Foo E.** 2018. Gibberellins promote nodule organogenesis but inhibit the infection stages of nodulation. Journal of Experimental Botany 69, 2117–2130.

**The role of gibberellin (GA) in legume nodulation is controversial, being reported to both enhance and inhibit the process. Now, in an elegant, multi-faceted investigation on peas McAdam *et al.* (2018) clearly show that GA is needed for nodule formation but inhibits the initiation of infection by *Rhizobium*. The investigators used genotypes varying in GA biosynthesis and signal transduction, combined with GA and GA-biosynthesis inhibitor applications, as well as an ethylene receptor mutation. Extensive measurements include developmental morphology and anatomy, gene expression, hormone levels and nitrogen fixation.**

Nodulation at the whole-root level consists of at least two spatially separate programs: infection at the epidermis, and nodule organogenesis originating in the inner cortex. The process commences with the exchange of chemical signals between the epidermal root hair and the *Rhizobium* bacteria in the soil (Nelson and Sadowsky, 2015; Ibáñez *et al.*, 2017). The perception of compatible rhizobia-produced NOD-factors by the plant host induces physical changes that enable colonization. This includes root hair curling and infection-thread formation, with transmission of the bacteria from cell to cell in a membrane-bounded infection-thread. The bacteria finally take up residence in membrane-bounded vesicles in the cortex, leading to establishment of the nodule through cell division in the inner-cortical cell layers of the root. Nodule development and nitrogen fixation, like many plant processes, are influenced by plant hormones (Ferguson and Mathesius, 2014). Auxin and cytokinin (CK) are involved in nodule initiation, growth, differentiation and positioning. Auxin accumulation at the site of nodule initiation, regulated by auxin transporters in the cell membranes, is crucial to nodule development (Kohlen *et al.*, 2018). *Rhizobium* infection rapidly induces the up-regulation of several CK biosynthesis genes, leading to CK accumulation and response in the region of the root where nodulation takes place (Gamas *et al.*, 2017). Ethylene is generally considered a negative regulator of nodulation (Guinel, 2015).

The role of gibberellins (GAs) in legume nodulation is controversial. Rather like Janus (the Roman god of duality who is often presented with two opposite-facing faces), GAs are reported to both enhance and inhibit nodulation. The paper by Erin McAdam and colleagues takes another look at the regulation of nodulation in pea by GAs, using an innovative series of GA biosynthesis and signal-transduction mutants (McAdam *et al.*, 2018). Reid’s group has for forty years been a leader in the use of genetics to elucidate the hormonal regulation of growth in peas, especially with regard to GAs, and they were the first to demonstrate that the growth-active GA in peas is GA_1_ (Reid *et al.*, 2010). One notable advantage of pea is that it is the only legume for which an extensive array of GA-biosynthesis mutants and signal-transduction mutants is available (see Box 1), most generated by Reid’s group. These include mutants with blocks at known points of the GA-biosynthesis pathway, as well as signal- transduction mutants (Weston *et al.*, 2008), so that both the GA levels and the signal-transduction pathway can be manipulated separately or together. This allows the examination of the effects of both GA levels and signalling on the full range of processes from infection through to mature nodules. Notable amongst the GA-biosynthesis mutants is a tiny pea mutant named *nana* (gene *na*) with internodes only a few millimetres long; *nana* has a block in the three-step conversion of *ent*-kaurenoic acid to GA_12_ (Reid *et al.*, 2010). The block in the DELLA negative-signal transduction pathway occasioned by the mutant genes *la* and *cry-s* results in an ultra-tall, skinny, light-green pea plant nicknamed ‘slender’, regardless of the presence of any mutations in the GA-biosynthesis pathway or the endogenous levels of GAs (DELLA signal-transduction proteins are so-named because they have a conserved N-terminal domain DELLA, i.e. Aspartate-Glutamate-Leucine-Leucine-Alanine). The root growth in *na* is only about 40% of wild type, yet the root growth of the DELLA mutants is similar to wild type regardless of the presence of *na* (Silva and Davies, 2007).

**Box 1. The role of pea in plant hormone research: a historical perspective**

Pea (*Pisum sativum*) has been central to much hormone research, including that of auxin and GAs, and has been used in bioassays for auxin, gibberellins and ethylene (Yopp *et al.*, 1986). The discovery of auxin was aided by structurally simple oat coleoptiles before work progressed to whole plants such as pea. [A wonderful historical account of initial efforts to determine the chemical nature of auxin has been told by Sam Wildman (1997).] A problem came when auxin applications to intact stems could not replicate the positive effects on elongation obtained with pea stem segments, leading to the erroneous concept that auxin was inactive in the growth of intact stems. It turns out that in a complex tissue applied auxin can have both positive and negative competing effects, determined by its location and concentration; intact stems do respond to exogenous auxin if the auxin is supplied in a continuous low dose, mimicking the natural transport from the stem apex (Yang *et al.*, 1993).

In the case of GAs the early findings derived from spraying whole plants with gibberellic acid (GA_3_) from fungal cultures (Phinney, 1983). GAs are now known to be a family of over 100 compounds, although most of these are inactive, being part of the biosynthetic or deactivation pathway (Sponsel and Hedden, 2010). An experimental advantage for GAs is that plants that are in any way GA-deficient, including dwarf peas, respond strongly to commercially available GA_3_, and plants that make their own GAs can be dwarfed by GA-biosynthesis inhibitors such as paclobutrazol. Some initially puzzling results using the slender pea mutant (genotype *la cry-s*) (Weston *et al.*, 2008) were the first indications of the DELLA proteins, transcriptional regulators that repress GA responses. Mutations in these proteins can produce positive or negative growth effects depending on whether the mutation is located in the regulatory or functional domain (Sun, 2010).

In 1980 Jonathan Goldthwaite suggested to me over a beer that all plant physiologists should concentrate on pea. Of course Arabidopsis was later chosen for its clear advantages, yet pea endures despite its large genome and transformation difficulties. Pea actually has significant advantages: its larger size and cauline structure permits experimental manipulation, and its genetics, starting of course with Gregor Mendel, are well known. Mendel’s tallness gene turned out to encode GA-3β-hydroxylase (oxidase), which is involved in the conversion of inactive GA_20_ to growth-active GA_1_ (Lester *et al.*, 1997). Being a legume pea also nodulates in association with *Rhizobium* bacteria to enable nitrogen fixation; legumes such as pea are a crucial lynchpin of many agricultural systems (Smýkal *et al.*, 2012; Smýkal *et al.*, 2016; see also Considine *et al.*, 2017, introducing the *Journal of Experimental Botany* special issue ‘Legumes, food security and climate change’). Indeed, when it comes to investigating the role of plant hormones in nodulation, pea has the largest and best characterized range of plant hormone mutants of any legume species. While the pea genome has yet to be fully sequenced (Kulaeva *et al.*, 2017) it is the subject of a sequencing effort (see www.france-genomique.org). Nonetheless the homologue of almost any Arabidopsis gene can now be isolated and characterized in pea, and the use of related genomes such as *Medicago* have overcome many of the limitations.

In light of such initial confusion in how plant hormones operate it is not surprising that there has been some controversy on the role of hormones in legume nodulation involving the interaction of two different organisms in a complex tissue, including several steps leading to a brand new plant organ encasing a complex mutualistic biochemistry. Pea is again an ideal plant for these investigations.

## Gibberellins in nodulation

Previous legume mutant/GA application studies have clearly suggested both a positive and a negative role for GA in nodulation. Pea mutants possessing root systems deficient in GAs exhibited a reduction in nodule organogenesis, and application of GA to the roots of GA-deficient *na* plants completely restored their number of nodules to that of the wild type (Ferguson *et al.*, 2005). Grafting studies also revealed that a wild-type shoot or root also restored the nodule number of a GA-deficient mutant. The role of GA does not, however, seem straightforward. Double mutants with *na* and a supernodulating allele still nodulated, but the nodule structures were aberrant; this indicated that severely reduced GA concentrations are not entirely inhibitory to nodule initiation, but that higher GA concentrations are required for proper nodule development. However, constitutive GA signalling mutants (*la cry-s*), whether with *NA* or *na*, also formed fewer nodules than wild-type plants, suggesting that an optimum degree of GA signalling is needed for nodule formation and that the GA signal, rather than the concentration of GA, is important for nodulation (Ferguson *et al.*, 2011). These findings appeared to be in conflict with recent findings in *Lotus* and *Medicago* using DELLA mutants and protein studies that suggested only a negative role for GA in nodule formation (Fonouni-Farde *et al.*, 2016; Jin *et al.*, 2016). GA application to wild-type plants in these species also suppressed nodule number. NOD-factor-activated expression of transcription factors and downstream early nodulation genes was suppressed by pre-treatment of wild-type *Lotus* and *Medicago* with GA, and also in the absence of GA treatment in *Medicago della* mutant lines. These studies provided molecular and physical evidence that GA inhibits nodulation events occurring at the epidermis and/or early in the nodulation process such as infection-thread formation.

McAdam *et al.* (2018) have resolved this paradox using an elegant series of pea GA mutants, with attention to the different stages and locations of the *Rhizobium* infection and nodule formation processes. It is now evident that GA has an opposing role in different cell layers of the root, suppressing events leading to infection-thread formation in the epidermis, but promoting nodule organogenesis in the inner cortex. In order to visualize the infection process the investigators used l*acZ*-labelled *Rhizobium*, which showed a dramatic increase in the number of infection-threads formed in GA-deficient *na* mutants. Yet despite increased root infection, *na* mutants often formed no nodules. Another striking feature of *na* mutants was that a proportion of infection-threads went on to form ramified structures within the root cortex. These highly ramified infection-threads were never associated with cell division or differentiation characteristic of nodules, showing that GA plays an important role in the checkpoint between infection and nodule organogenesis. All these differences in *na* plants were substantially reversed by the addition of exogenous GA_3_ to *na* plants. On the other side of the coin infection-thread formation was reduced in DELLA-deficient *la cry-s* GA-signalling pea mutants compared with wild-type plants. Moreover the number of infection-threads and ramification structures formed in GA- and DELLA-deficient *na la cry-s* plants was no different from DELLA-deficient *la cry-s* mutants, showing that the important factor is the GA-action pathway rather than the level of GA itself.

Nodule organogenesis involves de-differentiation, division, expansion and re-differentiation of inner cortical cells, into which the bacteria from infection-threads enter and ultimately fix nitrogen. The suppression of nodule size in *na* plants could be mimicked in wild-type peas by the addition of the GA-biosynthesis inhibitor paclobutrazol, and partially rescued in *na* mutants by addition of GA_3_. The expression of several key genes was significantly lower in the nodules of *na* plants, which have small or inactive nodules, compared to wild-type plants. Nitrogen fixation (estimated using the acetylene-reductase assay) was reduced by about 80% in the presence of *na*, showing that GA is required not only for nodule development but also for nodules to develop into nitrogen-fixing organs. Although the *la cry-s* (*della*) mutants produce fewer nodules than wild-type plants, the acetylene reductase rate of these nodules was not significantly different from nodules on wild-type plants, regardless of the presence or absence of *na*. This also shows that the effect of *na* on nodule function is entirely mediated through the DELLA proteins.

As ethylene is implicated as a possible intermediate in the action of GA in nodule formation (Foo *et al.*, 2016), McAdam *et al.* generated plants with a block in GA biosynthesis combined with defective ethylene perception. GA-deficient *na* mutants produce more ethylene and this appears to contribute at least in part to the low nodule number in this mutant. Disruption of ethylene perception, through the *ein2* mutation, elevated nodule number in *na ein2* plants to the same extent as that seen in *NA* plants. The expression of many of the genes was different in *na ein2* nodules compared with wild-type or *ein2* nodules. It appears that GA suppresses infection-thread formation relatively independently of ethylene, but acts partly through ethylene to suppress the transition from infection-thread to nodule initiation.

Overall we can conclude that GA, acting through DELLA proteins, suppresses infection-thread formation, but also acts through DELLAs to promote nodule organogenesis and the ultimate function of nodules as nitrogen-fixing organs. A diagrammatic scheme for the action of GA in pea nodulation is shown in Box 2.

**Box 2. Stages of nodulation and the roles of GA**

A diagrammatic cross section of pea root is shown with decreasing magnification moving clockwise to highlight different stages of nodulation and the roles of GA and other hormones in the process.

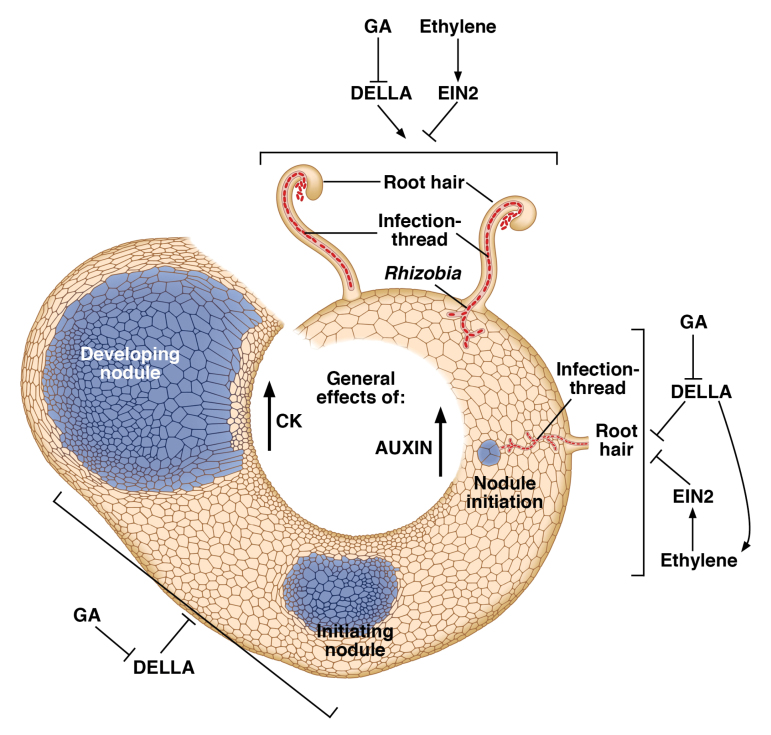

## Future directions

Researchers now have a sophisticated understanding of the early signalling events triggered by perception of rhizobial signals at the epidermis, and know that much of this pathway overlaps with signalling required for mycorrhizal symbioses (Geurts *et al.*, 2016). However, we know much less about the downstream genes and signals specifically involved in nodule organogenesis, and whether the differences between the different stages of nodulation are regulated by position or functional stage. Indeed GA clearly plays a negative role in mycorrhizal infection (e.g. Foo *et al.*, 2013), indicating GA as a potential differential regulator of the two symbioses in legumes. The results of McAdam *et al.* highlight several clear areas for future nodulation research. It would be interesting to examine the role of GA in different forms of nodulation (determinate versus indeterminate nodules) and actinorhizal species (non-legumes that form symbioses with N-fixing *Frankia* bacteria). Indeed, recent studies have shown that the *Rhizobia* that associate with determinate, but not indeterminate, nodulators themselves synthesize GA (Tatsukami and Ueda, 2016). The connection between GA, auxin and CKs during nodule organogenesis is also worthy of attention, especially given that GA and auxin interact in other processes such as stem elongation (Yang *et al.*, 1996). Future work may explore targets of these three key hormones and potential interactions. Related to this, the nature of the signal(s) that co-ordinates the (initially) spatially separated events of infection at the epidermis and concomitant activation of cell division and differentiation in the inner cortex is still unclear. It is possible that hormones, including GA, play a role in this communication process.

In conclusion I am reminded of a song on photosynthesis from the ‘Biochemists’ Songbook’ [by Harold Baum (1982), to the tune of ‘Auld Lang Syne’ no less; audio link at http://web.csulb.edu/~cohlberg/songbook.html], when it gets to the Calvin cycle: ‘And now occurs a jolly dance’. Clearly the hormonal regulation of nodule formation represents a jolly dance, and this one has two partners!
